# Evaluation of modelling study shows limits of COVID-19 importing risk simulations in sub-Saharan Africa

**DOI:** 10.1017/S095026882000120X

**Published:** 2020-06-09

**Authors:** T. Miyachi, T. Tanimoto, M. Kami

**Affiliations:** Medical Governance Research Institute, Tokyo, Japan

**Keywords:** COVID-19, mathematical modelling

## Abstract

Mathematical modelling studies predicting the spread of the coronavirus disease 2019 (COVID-19) have been used worldwide, but precisions are limited. Thus, continuous evaluation of the modelling studies is crucial. We investigated situations of virus importation in sub-Saharan Africa (SSA) to assess effectiveness of a modelling study by Haider N *et al*. titled ‘Passengers’ destinations from China: low risk of novel coronavirus (2019-nCoV) transmission into Africa and South America’. We obtained epidemiological data of 2417 COVID-19 cases reported by 40 countries in SSA within 30 days of the first case confirmed in Nigeria on 27 February. Out of 442 cases which had travel history available, only one (0.2%) had a travel history to China. These findings underline the result of the model. However, the fact that there were numbers of imported cases from other regions shows the limits of the model. The limits could be attributed to the characteristics of the COVID-19 which is infectious even when the patients do not express any symptoms. Therefore, there is a profound need for all modelling researchers to take asymptomatic cases into account when they establish modelling studies.

To the Editor-in-Chief:

The recently published article of Haider N *et al*., titled ‘Passengers’ destinations from China: low risk of novel coronavirus (2019-nCoV) transmission into Africa and South America’ is of great help for international organisations and national health authorities to improve preventative measurements against the virus importation. Haider N *et al*. concluded that risk of the virus importation in Africa was relatively low except South Africa, Ethiopia and Mauritius based on direct flights from four major cities of China (Wuhan, Beijing, Shanghai and Guangzhou) to the passengers' destination countries [1].

Considering the current situation, we would like to discuss more on their implications but also the challenges and effectiveness of modelling studies predicting the spread of the virus.

Since the COVID-19 emerged in China, mathematical modelling studies have been used worldwide. These help guide governments to form public health strategies. However, precisions are limited because they simplify complex social characteristics [2]. Few analyses were carried out to evaluate modelling studies.

In sub-Saharan Africa (SSA), precise models are particularly important to enhance their public health measurements and minimise the economic damage since their resources to combat diseases are already overwhelmed [3]. Thus, we investigated situations of virus importation in SSA to assess effectiveness of a modelling study on global transmission of COVID-19 by Haider N *et al*.

We obtained epidemiological data of 2417 COVID-19 cases reported by 40 countries in SSA within the 30 days of the first case confirmed in Nigeria on 27 February 2020 [4]. Out of 442 cases which had travel history available, Europe was the highest, 292 cases (66.1%), followed by Middle East 71 (16%), North America 32 (7.2%), Africa 28 (6.3%), Asia 16 (3.6%) and others 3 (0.6%).Concerning Asia, only one had a travel history to China, which was reported in Somalia on 16 March 2020.

These results partially supported the modelling study since there was only one case from China in SSA. However, the fact that there were numbers of imported cases from other regions shows the limits of the model. The limits could be attributed to the characteristics of the COVID-19, which is infectious even when the patients do not express any symptoms [5]. Undetected cases might have already spread across the world by the time importation risk of COVID-19 from China was simulated.

Consequently, there is a profound need for all modelling researchers to take asymptomatic cases into account when they establish modelling studies. Our study involving countries lacking some data is too small and too insufficient to make a definite conclusion. Further studies are warranted.
